# Cerebrospinal fluid light and heavy neurofilament level increased in anti‐*N*‐methyl‐d‐aspartate receptor encephalitis

**DOI:** 10.1002/brb3.1354

**Published:** 2019-07-17

**Authors:** Jiayu Li, Yong Gu, Hongwei An, Zheyi Zhou, Dong Zheng, Zhanhang Wang, Zehuai Wen, Hai‐Ying Shen, Qi Wang, Honghao Wang

**Affiliations:** ^1^ Department of Neurology, Nanfang Hospital Southern Medical University Guangzhou China; ^2^ Institute of Clinical Pharmacology Guangzhou University of Chinese Medicine Guangzhou China; ^3^ Department of Encephalopathy Hainan Provincial Hospital of Traditional Chinese Medicine Haikou China; ^4^ Department of Neurology Liuzhou Traditional Chinese Medical Hospital Liuzhou China; ^5^ Department of Neurology The Affiliated Brain Hospital of Guangzhou Medical University Guangzhou China; ^6^ Department of Neurology 999 Brain Hospital Guangzhou China; ^7^ RS Dow Neurobiology Laboratories Legacy Research Institute Portland Oregon

**Keywords:** anti‐NMDAR encephalitis, cerebrospinal fluid, cytokine, modified Rankin scale, neurofilament heavy subunit, neurofilament light subunit

## Abstract

**Background:**

Neurofilaments (Nf) are a series of highly specific scaffolding proteins of neurons. Neurofilament light chains (Nf‐L) and the heavy one (Nf‐H) are subunits of Nf, and they are recognized as potent productions of neural damage. The concentrations of Nf aggrandized significantly in neurological disease including neuromyelitis optica, multiple sclerosis, and Alzheimer's disease. However, whether Nf in cerebrospinal fluid (CSF) elevated in anti‐*N*‐methyl‐d‐aspartate receptor (NMDAR) encephalitis is unclear. Here, we aimed to detect whether CSF Nf is altered in NMDAR and whether changes in CSF Nf can serve as an objective and effective biomarker to evaluate disease severity and prognosis.

**Methods:**

We collected 24 anti‐NMDAR encephalitis patients, 11 viral meningoencephalitis/encephalitis (VM) patients, and 21 controls in this study. CSF Nf‐L, Nf‐H, and cytokine levels (IL‐1β, IL‐6, and IL‐17A) were determined by enzyme‐linked immunosorbent assay (ELISA) and compared between groups. We evaluated patients’ clinical outcomes or prognosis according to modified Rankin scale (mRS) score.

**Results:**

Compared with controls, both CSF Nf‐L and Nf‐H levels were significantly increased in anti‐NMDAR encephalitis patients. While compared with VM patients, only Nf‐L were increased in anti‐NMDAR encephalitis patients. Moreover, CSF Nf‐L were positively correlated with concentration of cytokines (IL‐1β, IL‐17A) and mRS scores in anti‐NMDAR encephalitis patients. After treatment, both CSF Nf‐L and Nf‐H levels decreased. Furthermore, the Nf‐L during follow‐up positively correlated with 3‐month mRS scores, and ΔNf‐L positively correlated with ΔmRS.

**Conclusions:**

Briefly, CSF Nf‐L levels notably increased in anti‐NMDAR encephalitis patients in acute phase and positively correlated with disease severity. It could be considered as a useful indicator for clinical outcomes and prognosis.

## 1 INTRODUCTION

Anti‐*N*‐methyl‐d‐aspartate receptor (NMDAR) encephalitis is commonly known as an autoimmune disease with characterized symptoms as psychosis, epilepsy, autonomic nervous system in dysfunction, and various disturbances in movement (Dalmau, Lancaster, Martinez‐Hernandez, Rosenfeld, & Balice‐Gordon, 2011; Kayser, Titulaer, Gresa‐Arribas, & Dalmau, 2013). The concept of anti‐NMDAR encephalitis associated with ovarian teratoma was firstly recorded in 2007; twelve women with a series of neuropsychiatric syndrome were detected to have autoantibodies binding to glutamate receptors (type NMDA) (Dalmau et al., 2007). This clinical phenotype was due to internalization of NMDARs mediated by anti‐NMDAR antibodies. However, anti‐NMDAR encephalitis was subsequently found in patients without tumors. Thus, it is possible that such antibodies could arise from immunologic triggers else instead of tumor (Dalmau et al., 2008). Though B and T leukocytes were proposed to exert in anti‐NMDAR encephalitis, further research is needed to illuminate the underlying immunopathogenesis of this disease (Tuzun et al., 2009).

Neurofilaments (Nf) are a series of highly specific scaffolding proteins of neurons. They are located in axons and contain three structurally similar subunits named as Nf‐heavy chains (Nf‐H), Nf‐medium chains (Nf‐M), and Nf‐light chains (Nf‐L) with molecular weight of 200, 150, and 68 kDa, respectively (Petzold, 2005). The disruption of Nf occurs under the conditions of neurodegeneration or neuronal cell death (Gaiottino et al., 2013; Petzold, 2005); subsequently, Nf increase in cerebrospinal fluid (CSF) or serum (Petzold et al., 2011; Zetterberg et al., 2013). Nf‐L are major structural subunits in myelinated axons, and they are essential for the formation of heteropolymer by copolymerization with two other subunits, Nf‐M and Nf‐H (Geisler & Weber, 1981; Lee et al., 1993). These subunits play a significant role in maintaining axonal caliber and conduction velocity. Deficiency of Nf‐L could influence the morphological integrity of axon and effective conduction of nerve impulses. Absence of Nf‐H subunit does not greatly reduce the neurofilaments in peripheral and sensory axons, but it is vital to the survival of motor and sensory axons. Increased Nf‐L levels were found in neurological diseases including human spinal cord injury (Kuhle, Gaiottino, et al., 2015), multiple sclerosis, and various neurodegenerative diseases (Kuhle, Disanto, et al., 2015; Petzold, 2005; Sjogren et al., 2000). Likewise, Nf‐H have been reported to increase in acute ischemic stroke (Singh et al., 2011). The levels of Nf‐L and Nf‐H in CSF are taken for more indicative of brain pathology than those in serum. However, whether CSF Nf‐L and Nf‐H elevated in anti‐NMDAR encephalitis patients remains undefined. CD4^+^ T cells take part in pathogenesis and development in some autoimmune diseases, such as multiple sclerosis and rheumatoid arthritis (Damsker, Hansen, & Caspi, 2010). In anti‐NMDAR encephalitis, the immune response is probably triggered initially by tumor, virus, or uncertain precipitating factors else and subsequently activated and amplified in the CNS (Dalmau et al., 2011; Tuzun et al., 2009; Zandi et al., 2015). Here, we measured the levels of cytokines that are associated with the function of these cells, including IL‐1β, IL‐6, and IL‐17A. Previously, we have reported other high expression inflammatory cytokines in serum or CSF in anti‐NMDAR encephalitis patients (Ai et al., 2018; Patakas et al., 2012).

We aimed to investigate whether Nf‐L or Nf‐H levels were altered in anti‐NMDAR encephalitis patients and to further explore whether the Nf proteins were associated with disease severity, clinical outcomes, and prognosis. Considering that another major encephalitis in central nervous system is caused by virus, we compared these indicators in anti‐NMDAR encephalitis patients with patients in control group and patients with viral meningoencephalitis/encephalitis (VM).

## 2 MATERIALS AND METHOD

### 2.1 Patients groups and control group

Our study included three groups—anti‐NMDAR encephalitis group (24 patients); viral meningoencephalitis/encephalitis (VM) group (11 patients); and control group (21 patients). All of them were selected from the Department of Neurology, Nanfang Hospital of Southern Medical University. We adopted the revised criteria defined by Graus et al. in 2016 to elect the anti‐NMDAR encephalitis patients (Graus et al., 2016). Viral meningoencephalitis/encephalitis is caused by a variety of viruses and belongs to the category of etiological diagnosis. Herpes simplex virus, adenovirus, and enterovirus are common pathogens. The diagnostic criteria of the viral meningoencephalitis are as follows: (a) symptoms of acute onset of systemic infection poisoning (fever, headache); (b) meningeal irritation sign; (c) CSF lymphocytes are moderately elevated, leukocyte count 10–300 (×10^6^/L), glucose 2.5–3.5 mM, protein < 0.8 g/L; (d) cerebrospinal fluid pathology, no evidence of bacterial (including tuberculosis and mycosis) infection was found; and (e) exclude other diseases. The diagnostic criteria of the viral encephalitis are as follows: (a) prodrome of viral infection. Subsequently, high fever, severe headache, nausea, vomiting, and disturbance of consciousness often accompanied by focal nervous system positioning signs and convulsions (convulsions can be the first symptoms); (b) clinically, there are signs of brain parenchyma damage caused by virus infection; (c) cerebrospinal fluid showed or showed no inflammatory changes, and no evidence of bacterial (including tuberculosis and mycosis) infection was found; (d) the EEG showed diffuse abnormality (some could be focal), and no sign of space occupying lesion in CT or MR; (e) serum antibody titer increased significantly. Patients in VM group were identified according to criteria above, and they were excluded from other types of CNS diseases. The age‐ and sex‐matched control individuals were patients with other neurological disorders, including movement disorders and cerebrovascular diseases, among whose clinical symptoms and laboratory examinations supported neither for a diagnosis of anti‐NMDAR encephalitis nor viral meningoencephalitis/encephalitis. Anti‐NMDAR antibodies were found to be positive in all CSF of the anti‐NMDAR encephalitis patients selected at the time of diagnosis by cell‐based analysis. We assessed their dominating clinical outcomes and prognosis on the ground of the modified Rankin scale (mRS).

### 2.2 Ethics statement

All procedures were carried out with the adequate understanding and written consent of the participants as well as the approval of the Ethics Committee of the Nanfang Hospital, Southern Medical University.

### 2.3 Biochemical assays

All of the CSF samples were collected by the time of diagnoses and reserved in the refrigerator at −80°C until we performed the assays. The Nf‐L (IBL) and phosphorylated neurofilament (pNf‐H; Biovendor) were quantified by Sandwich ELISA immunosorbent assay according to the manufacturer's instructions. The concentrations of IL‐1β, IL‐6, and IL‐17A were determined by enzyme‐linked immunosorbent assays (Bender MedSystems GmbH Campus Vienna).

### 2.4 Follow‐up evaluations

Nine of the 24 anti‐NMDAR encephalitis patients were re‐examined those same parameters in our hospital in 3 months later after onset, as routine follow‐up. The other 15 patients did not come back to our hospital or rejected the repeated lumbar puncture examination. Follow‐up patients’ neurological outcomes were assessed two times by mRS when they got the worst conditions (Max mRS score) as well as 3 months after ictus (3 months mRS score). ΔNf‐L = Nf‐L (acute stage)‐Nf‐L (follow‐up), ΔIL‐1β, ΔIL‐6, ΔIL‐17A, and ΔmRS were similar to this calculation.

### 2.5 Statistical analysis of data

We carried out all statistical analysis by SPSS version 20.0 (IBM Corp). CSF Nf‐L, pNf‐H, IL‐1β, IL‐6, IL‐17A, and ages were presented in the form of the mean ± standard deviation (*SD*) for the data distributed normally. The information of mRS scores was demonstrated by medians (min, max) when the data were not normally distributed. One‐way ANOVA was adopted to distinguish the differences about Nf‐L, pNf‐H, and cytokines between groups, paired sample *t* test to analyze those parameters of nine follow‐up anti‐NMDAR encephalitis patients during acute stage and follow‐up. Pearson test was used to assess the correlations between Nf and cytokines, while Spearman's correlations to analyze the Nf and mRS scores. We decided the sample size by the statistical sample size formula *n* = [(μα + μβ)2 (1 + 1/K)σ2]/δ2 using the online sample size statistics tool (http://powerandsamplesize.com/). A *p* value < 0.05 was viewed as an indication of statistical significance in the actual study.

## 3 RESULTS

### 3.1 Clinical characteristics and demographic data of participants

The baseline characteristics of the anti‐NMDAR encephalitis group, VM group, and controls were shown (Table 1). The mean ages (years) of the anti‐NMDAR encephalitis patients were 34.02 ± 18.71, the VM group 33.82 ± 16.38, and the control group 36.48 ± 15.46. The median Max mRS score and 3 months mRS score of the anti‐NMDAR encephalitis group was 4.0 (range 3.0–5.0) and 3.0 (range 1.0–5.0), respectively. Three of the 24 anti‐NMDAR encephalitis patients (12.5%) had ovarian teratoma. There were no statistical significances in age and sex distribution among the three groups.

**Table 1:** Clinical features of patients and control individuals

	anti‐NMDAR encephalitis (*n* = 24)	VM (*n* = 11)	Controls (*n* = 21)
Gender, female/male	14/10	4/7	10/11
Age (years)	34.02 ± 18.71	33.82 ± 16.38	36.48 ± 15.46
CSF IL‐1β (pg/ml, mean ± *SD*)	5.73 ± 3.50 ****	3.74 ± 1.98	1.84 ± 0.94 ****
CSF IL‐6 (pg/ml, mean ± *SD*)	10.21 ± 5.78 ****	7.20 ± 6.52	3.81 ± 1.47 ****
CSF IL‐17A (pg/ml, mean ± *SD*)	10.29 ± 5.42 ****	4.62 ± 1.15	2.82 ± 1.67 ****
Symptom (*n*, %)			
Prodromal symptoms (fever)	13 (54.2)	8 (72.7)	_
Disorder of behavior and cognition	21 (87.5)	3 (27.3)	_
Memory deficits	12 (50.0)	1 (9.0)	_
Speech disturbances	14 (58.3)	1 (9.0)	_
Seizures	18 (75.0)	3 (27.3)	_
Movement disorders	11 (45.8)	2 (18.2)	_
Loss of consciousness	15 (62.5)	6 (54.5)	_
Autonomic nerve disorder	10 (41.7)	0 (0.0)	_
Hypopnea	8 (33.3)	2 (18.2)	_
Anti‐NMDAR antibody	24	0	0
Max mRS score (median, range)	4.0 (3.0–5.0)	_	_
3‐month mRS score (median, range)	3.0 (1.0–5.0)		

Abbreviations: anti‐NMDAR encephalitis, anti‐*N*‐methyl‐d‐aspartate receptor encephalitis; CSF, cerebrospinal fluid; EEG, electroencephalogram; VM, viral meningoencephalitis/encephalitis.

* *p* < 0.05.

** *p* < 0.01.

*** *p* < 0.001.

**** *p* < 0.0001.

### 3.2 Nf‐L, pNf‐H, and cytokines in patients group and control group

**Figure 1:**
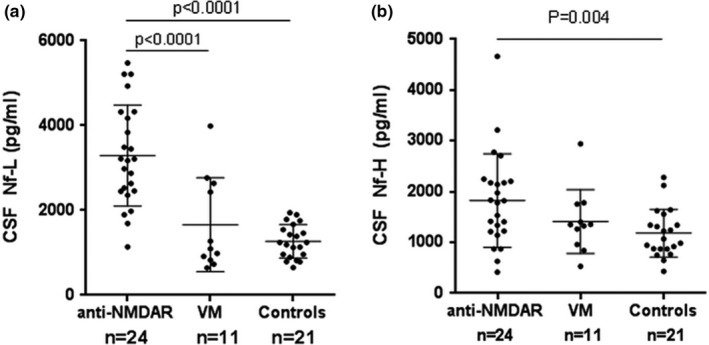
Comparison of Nf‐Land pNf‐H levels between patients and control. (a) CSF Nf‐L levels were higher in anti‐NMDAR encephalitis patients compared with VM and controls (*p* < 0.0001). (b) CSF pNf‐H levels were increased in anti‐NMDAR encephalitis patients compared with controls (*p* = 0.004)

Means of CSF Nf‐L were 3,292.63 pg/ml for anti‐NMDAR encephalitis group, 1,662.27 pg/ml for VM group, and 1,270.57 pg/ml for control group. Means of CSF pNf‐H for anti‐NMDAR encephalitis patients, VM patients, and controls were 1824.63, 1,411.09, and 1,182.57 pg/ml, respectively. Compared with controls, Nf‐L (*p* < 0.0001) and pNf‐H (*p* = 0.004) in anti‐NMDAR encephalitis group were increased, while both Nf‐L and pNf‐H levels in VM group showed no statistical differences. CSF Nf‐L level but not pNf‐H showed statistical differences between the VM group and patients with anti‐NMDAR encephalitis (Figure 1a,b). Concentrations of IL‐1β, IL‐6, and IL‐17A dramatically increased in anti‐NMDAR encephalitis group compared with controls (*p* < 0.0001) (Table 1).

### 3.3 Positive correlations between Nf, cytokines, and mRS score

An analysis of relationships between Nf, cytokines, and mRS score was conducted (Table 2). In anti‐NMDAR encephalitis group, Nf‐L were positively correlated with IL‐1β (*p* = 0.017), IL‐17A (*p* = 0.0001), and Max mRS scores (*p* = 0.020), respectively, while pNf‐H had no correlations with any cytokines or mRS scores in both groups (data not shown).

**Table 2:** Relationship between CSF Nf levels and cytokines or mRS scores in NMDAR patients

	*p* Value	*r* Value	
Nf‐L			
CSF IL‐1β	0.017	0.436	
CSF IL‐6	0.124	0.245	
CSF IL‐17A	0.0001	0.676	
Max mRS scores	0.020	0.422	
pNf‐H		
CSF IL‐1β	0.065	0.371	
CSF IL‐6	0.088	0.268	
CSF IL‐17A	0.167	0.206	
Max mRS scores	0.219	0.166	

Abbreviation: mRS, modified Rankin scale.

### 3.4 Follow‐up measurement of 9 NMDAR patients

**Figure 2:**
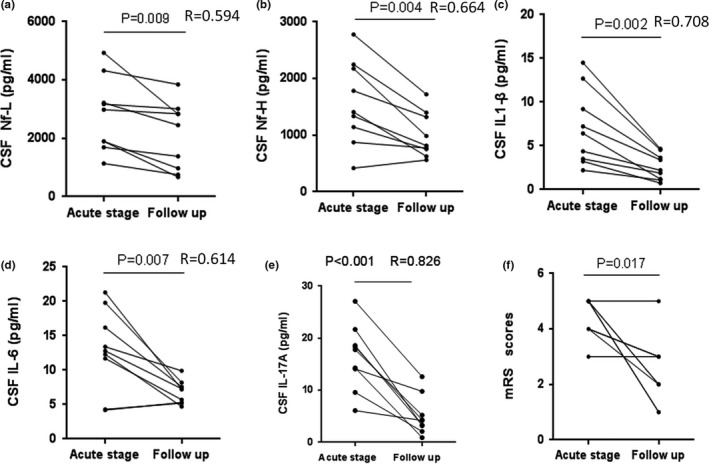
Follow‐up evaluation of clinical parameters in anti‐NMDAR encephalitis. CSF Nf‐L (a) and pNf‐H (b) levels were significantly decreased when followed up (Nf‐L, *p* = 0.009; Nf‐H, *p* = 0.004). IL‐1β (c) IL‐6 (d), IL‐17A (e), and mRS scores (f) were significantly decreased when followed up (IL‐1β, *p* = 0.002; IL‐6, *p* = 0.007; IL‐17A, *p* < 0.0001; mRS scores, *p* = 0.017)

**Figure 3:**
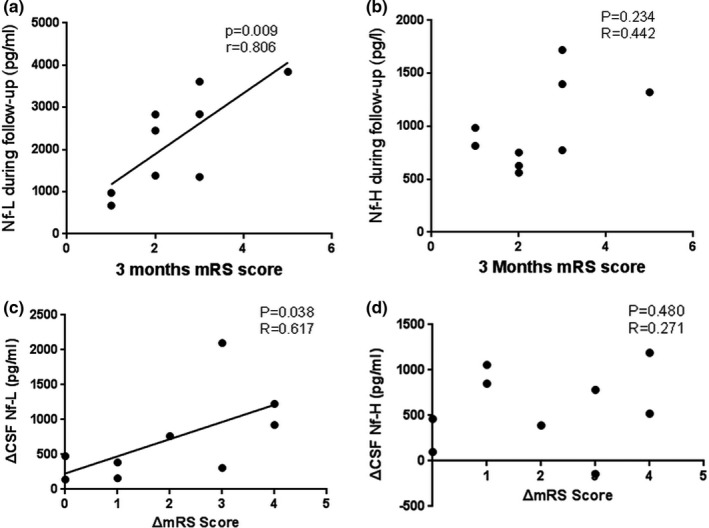
Correlation between Nf and mRS scores during follow‐up. There was a positive correlation between Nf‐L during follow‐up and 3–month‐ mRS (a) (*p* = 0.009), ΔNf‐L, and ΔmRS (c) (*p* = 0.038), respectively. There was no correlation between neither Nf‐H during follow‐up and 3‐month mRS (b) (*p* = 0.234), nor ΔmRS and the ΔpNf‐H (d) (*p* = 0.480)

As our results pointed out the relevance between Nf‐L and disease severity (mRS score) in anti‐NMDAR encephalitis patients in the acute phase, we further assessed whether CSF Nf, cytokines, and mRS score altered after therapy. Nine follow‐up anti‐NMDAR encephalitis patients (female:male; 6:3) received the remeasurement of these indexes. After treatment, CSF Nf‐L, pNf‐H, IL‐1β, IL‐6, IL‐17A, and mRS score decreased (Figure 2a–f). In addition, Nf‐L during follow‐up were associated with 3 months mRS scores (Figure 3a), and the ΔNf‐L is positively correlated with the ΔmRS (Figure 3c), while no such relations were found in Nf‐H (Figure 3b,d). The decrease of all cytokines has no relation with ΔNf‐L or ΔNf‐H (Table 3).

**Table 3:** Relationship between decrease of Nf levels and cytokines in NMDAR patients

	*p* Value	*r* Value
Δ Nf‐L		
Δ CSF IL‐1β	0.110	0.453
Δ CSF IL‐6	0.346	0.154
Δ CSF IL‐17A	0.064	0.547
Δ pNf‐H		
Δ CSF IL‐1β	0.089	0.492
Δ CSF IL‐6	0.128	0.424
Δ CSF IL‐17A	0.068	0.538

## 4 DISCUSSION

Our research discovered that CSF Nf‐L and pNf‐H increased in anti‐NMDAR encephalitis patients compared with controls. After treatment, CSF Nf‐L and pNf‐H decreased. While compared with VM patients, only CSF Nf‐L statistically increased in anti‐NMDAR encephalitis patients. Additionally, in anti‐NMDAR encephalitis patients, Nf‐L were relevant to cytokines (IL‐1β, IL‐17A) and mRS scores, and ΔmRS is positively correlated with ΔNf‐L.

Nf release resulting from neuronal damage (Gaiottino et al., 2013). Whereafter, Nf‐L concentrations elevate in the CSF and serum (Petzold et al., 2011; Zetterberg et al., 2013). In animal models, neurofilaments were verified to maintain the speed of signal transduction in nerve and the axonal caliber. Nf‐L knockout mice seemed to have smaller axon diameter (twofold to threefold reduction) and slower conduction speed (50% decrease) (Julien & Mushynski, 1998), which can lead to neurological dysfunctions. Besides, Nf‐L‐targeted disruption mice showed the feature of neurodegenerative diseases–protein aggregation in motoneurons (Li et al., 2006). Additionally, Axelsson et al have propounded the feasibility that Nf‐L polymerize by itself leading to protein accumulation and aggregate formation, which may concern with CNS diseases (Axelsson et al., 2014). Thus, Nf‐L was related with neurological diseases. In this study, we discovered that CSF Nf‐L were higher in anti‐NMDAR encephalitis patients and related with mRS scores, which implied the axonal injuries and Nf‐L accumulation in CSF. Similarly, increased Nf‐L has been discovered in neuromyelitis optica and multiple sclerosis (Madeddu et al., 2013; Wang et al., 2013). Thus, Nf‐L may be related with autoimmune encephalitis. Compared to Nf‐L, Nf‐H were found in higher level in anti‐NMDAR encephalitis patients only when compared with control group but not with VM group. The breakdown of Nf depends on protease digestion, and susceptibility of Nf to protease digestion decreases with increase of phosphorylation. Nf‐H is the most extensively phosphorylated protein of the human brain and pNf‐H was considered to show lower activities to proteolytic enzymes (Goldstein, Sternberger, & Sternberger, 1987). Nf‐L are more susceptible to protease activity than Nf‐H. What's more, Petzold A had proposed that Nf‐L are more sensitive to be detected than the heavy chain (Petzold, 2005). By comparison, Nf‐L are more suitable to mark brain damage and reflect the severity and outcomes of disease.

Immune B cells and T cells are indispensable regulators and effectors in immune responses to the formation and development of anti‐NMDAR encephalitis (Camdessanché et al., 2011; Chen et al., 2018; Hachiya et al., 2013; Peng et al., 2019). However, how they enter the CNS compartment or the blood–brain barrier is undefined. IL‐1β has been taken for a contributing factor in either autoinflammatory or autoimmune diseases and the induction of fever (Dinarello, 2011; Horai et al., 1998; Sims & Smith, 2010). It was found to damage cognitive abilities in Alzheimer's disease and influence the Th17 differentiation in naive T cells of mouse and human (Acosta‐Rodriguez, Napolitani, Lanzavecchia, & Sallusto, 2007; Bossu et al., 2007; Chung et al., 2009). Besides, inflammation and ischemic brain injury could be alleviated by IL‐1 receptor antagonist treatment in model rats (Pradillo et al., 2012). Elevated IL‐6 has been reported in autoimmune diseases like neuromyelitis optica (Uzawa et al., 2010). Th17 cells were demonstrated to increase in multiple sclerosis (Kebir et al., 2009). IL‐17A, a proinflammatory cytokine derived from Th17 cells, was reported to increase in anti‐NMDAR encephalitis patients (Liu et al., 2018). Consistently, our research revealed the increase of IL‐1β, IL‐6, and IL‐17A in anti‐NMDAR encephalitis patients compared with control. Moreover, Nf‐L positively correlated with cytokines (IL‐1β and IL‐17A) and mRS score, suggesting the possibility that Nf‐L concerned with inflammation reaction and the prognosis and severity in anti‐NMDAR encephalitis.

Interestingly, our results proved that after treatment, CSF Nf‐L, Nf‐H, cytokines, and mRS scores observably decreased in anti‐NMDAR encephalitis, indicating the effectiveness of treatment. Furthermore, the ΔmRS is positively correlated with ΔNf‐L. In other words, after treatment, the more the Nf‐L declined, the more obvious improvement of disease showed. It further proved that Nf‐L related with the disease severity.

We speculated that the decrease of CSF Nf‐L was potentially due to the catabatic inflammatory injuries in CNS after getting treatment. Alternatively, descending concentration of CSF Nf‐L could protect neuronal cell from inflammatory responses and injury. Though the underlying immunologic mechanism why Nf‐L altered is indeterminate, Nf‐L concentrations could play an important role in reflecting outcomes and prognosis of anti‐NMDAR encephalitis. Whether there is an effective way to repair neurofilament protein to treat cerebral lesion remained to be confirmed.

## 5 CONCLUSION

Briefly, our study discovered that anti‐NMDAR encephalitis patients get a noteworthy higher CSF Nf‐L and pNf‐H levels. And the increase of Nf‐L was positively related to disease severity, supporting that Nf‐L are more meaningful to reflect the potential pathogenesis of this autoimmune disease and indicate the clinical outcomes or prognosis. It would shed a new light to access disease severity, efficacy, and prognosis by using a biomarker in anti‐NMDAR encephalitis.

## CONFLICT OF INTEREST

None.

## DATA ACCESSIBILITY STATEMENT

The data that support the findings of this study are available from the corresponding author upon reasonable request.
